# An uncommon presentation and hybrid approach for the management of an unruptured 8 cm common iliac artery aneurysm

**DOI:** 10.1016/j.ijscr.2019.11.063

**Published:** 2019-12-09

**Authors:** Maria Asad, Krishna Venigalla, Muhammad Asad Rahi

**Affiliations:** a The School of Medical Sciences, Faculty of Biology, Medicine & Health, University of Manchester, Stopford Building, 99 Oxford Road, M13 9PG, United Kingdom; b Lancashire Teaching Hospital NHS Trust, Regional Vascular Centre, Royal Preston Hospital, Sharoe Green Ln, Fulwood, Preston, PR2 9HT, United Kingdom

**Keywords:** CT, Computed tomography, CVA, Cerebrovascular accident, ASA, American Society of Anesthesiologists, EVAR, Endovascular Aneurysm Repair, AUI, Aorto-Uni-Iliac, Aneurysm, Common Iliac Artery, EVAR, Hybrid

## Abstract

**Introduction:**

Common Iliac Artery Aneurysms are rare in the general population and can be difficult to diagnose.

**Presentation of case:**

An 82-year-old lady initially presented to her GP with non specific symptoms, but was found to have a right iliac fossa mass on examination. Initial Blood Tests and clinical observations were unremarkable. A CT scan demonstrated a large, 8 cm Isolated Right Common Iliac Aneurysm. She was admitted following the scan for monitoring and surgical intervention. In view of her mild frailty, she underwent a hybrid approach: Right Aorto-Uni-Iliac Stent Graft and Right to Left femoro-femoral crossover graft and right internal iliac artery embolization.

**Discussion:**

We discuss the rarity, variety of clinical presentations and aetiology of Common Iliac Artery Aneurysms. These aneurysms are at high risk of rupture and in the elective setting, many are repaired when they reach over 3.5 cm. With the advancement and preference of minimally invasive techniques, Endovascular Aneurysm Repair (EVAR) is replacing Open Surgery. In some instances, such as our case, the anatomy is unsuitable for a standalone EVAR procedure. Thus a hybrid operation was performed.

**Conclusion:**

Common Iliac Artery Aneurysms should be considered in a differential diagnosis of lower abdominal mass. If presenting in this manner, the aneurysm can be of significant size and at high risk of rupture, requiring urgent surgical intervention.

## Highlights

• Common Iliac Artery Aneurysms are rare and pose a diagnostic challenge.
• Surgical Intervention is usually required but is often high risk.
• Hybrid Procedure is a safer option for aneurysm repair in high risk patients.

## 1 Introduction

Isolated Iliac Artery Aneurysms (IIAA) are uncommon, accounting for 2–7% of all Intra Abdominal Aneurysms [1]. Iliac artery aneurysms can occur in conjunction with Abdominal Aortic Aneurysms (AAA) in up to 40 % of cases [2]. The majority (70 %) of IIAA involve the common iliac artery with only 20–25 % primarily affecting the internal iliac artery [1].

Like AAA, the IIAA carry a high risk of rupture on reaching a critical size. An Aneurysm of the Common Internal Iliac Artery (CIAA) is defined as being a permanent localised dilatation >1.5 cm in diameter. In our patient, the size of the aneurysm was over 5 times this definition.

The literature demonstrates a greater 5:1 male prevalence of IIAA, similar to that of AAA. This is likely due to the shared pathogenesis, primarily degeneration, but also includes inflammation and biomechanical wall stress. Underlying aetiology is usually atherosclerosis and less commonly trauma, infection, pregnancy and vascular collagen diseases [3].

The gold standard treatment for CIAA had been open surgical repair with prosthetic grafts. However, this can be technically challenging and the proximity of the aneurysm to other structures within the pelvis carries high risk to visceral, genitourinary and pelvic venous structures [4]. Less invasive, endovascular techniques are replacing open surgical repair but the anatomy of the aneurysm may not be suitable for standalone endovascular procedure.

We outline a hybrid technique utilised in a patient high risk for open intra-abdominal intervention. EVAR with AUI and fem-fem crossover graft surgery was opted as a safer alternative.

This case is reported in line with the SCARE criteria [5].

## 2 Case presentation

We present the case of an 82-year lady referred by her GP to secondary care services due to non specific abdominal pain, weight loss, appetite loss and the incidental finding of a non pulsatile, right iliac fossa mass on physical examination. She had a past medical history of medication managed hypertension, CVA, Osteopenia and was reasonably mobile and independent. She had mild frailty according to the Patient Frailty Score. She was an ex-trivial smoker (<1 a day) and consumed little alcohol.

She was referred to the local Colorectal Team under the suspected cancer pathway, therefore an US Abdomen was not requested. In the Colorectal clinic her blood tests were noted to be unremarkable; with a normal Full Blood Count, Renal function, Liver function and thyroid function. The abdominal mass was further described to be in the right iliac fossa, approximately 8−10 cm in diameter and partially mobile, interestingly only in the transverse plane.

**Fig. 1:**
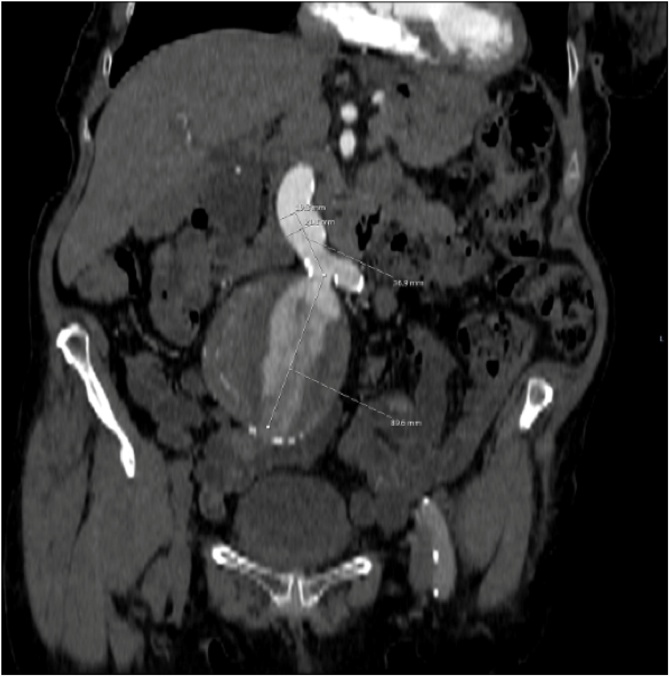
Coronal view, CT Abdomen Pelvis demonstrating normal Aorta and left Common Iliac Artery and Aneursymal Right Common Iliac Artery.

A CT Abdomen Pelvis was requested to further elucidate the unusual finding. This revealed an 8 cm Right Common Iliac Aneurysm (Fig. 1) with a patent right internal iliac artery. Importantly, the Abdominal Aorta diameter was of normal calibre. This lady was admitted following the scan and discussed in the Vascular Multi-Disciplinary Team Meeting. She remained clinically stable.

After Anaesthetic review, the patient was deemed to be an ASA Grade 3. She was planned for a combined vascular(hybrid) procedure, with vascular surgeons and interventional radiologists in a Hybrid Theatre. She underwent urgent Endovascular treatment by Right Aorto-Uni-Iliac Stent Graft and Right to Left femoro-femoral crossover graft and right internal iliac artery embolisation.

The initial step of the procedure entailed coil embolization of the Right Internal Iliac Artery with a variety of Nester coils and a 14 mm Amplatzer plug. The patient was also given 5000 IU of Heparin intra-operatively.

**Fig. 2:**
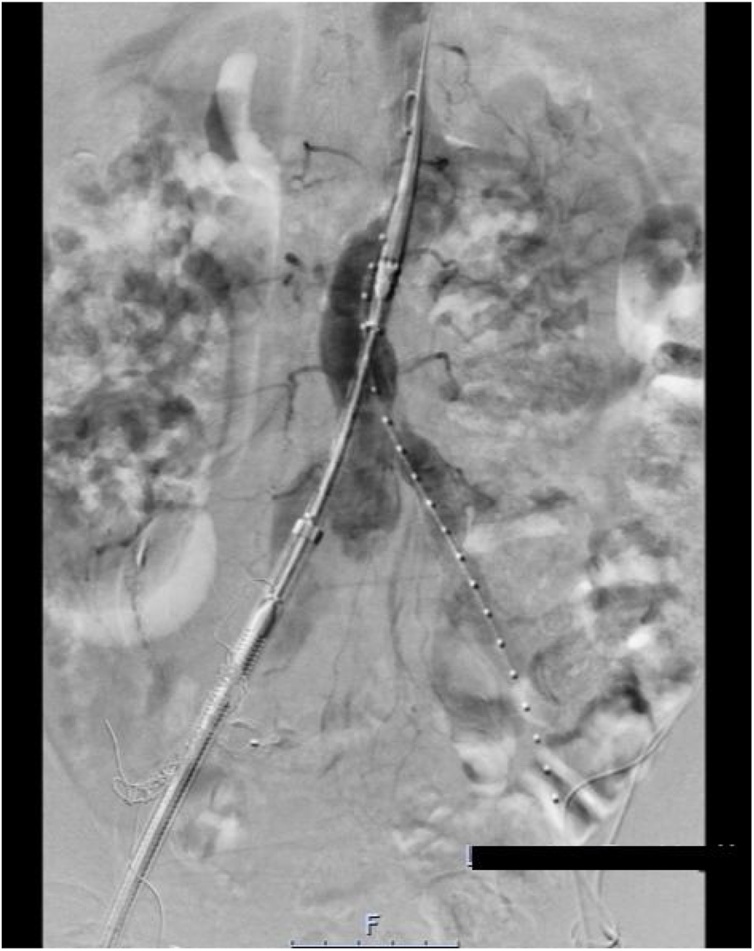
Initial Deployment of Aorto-uni-iliac device through Right Common Femoral Artery.

**Fig. 3:**
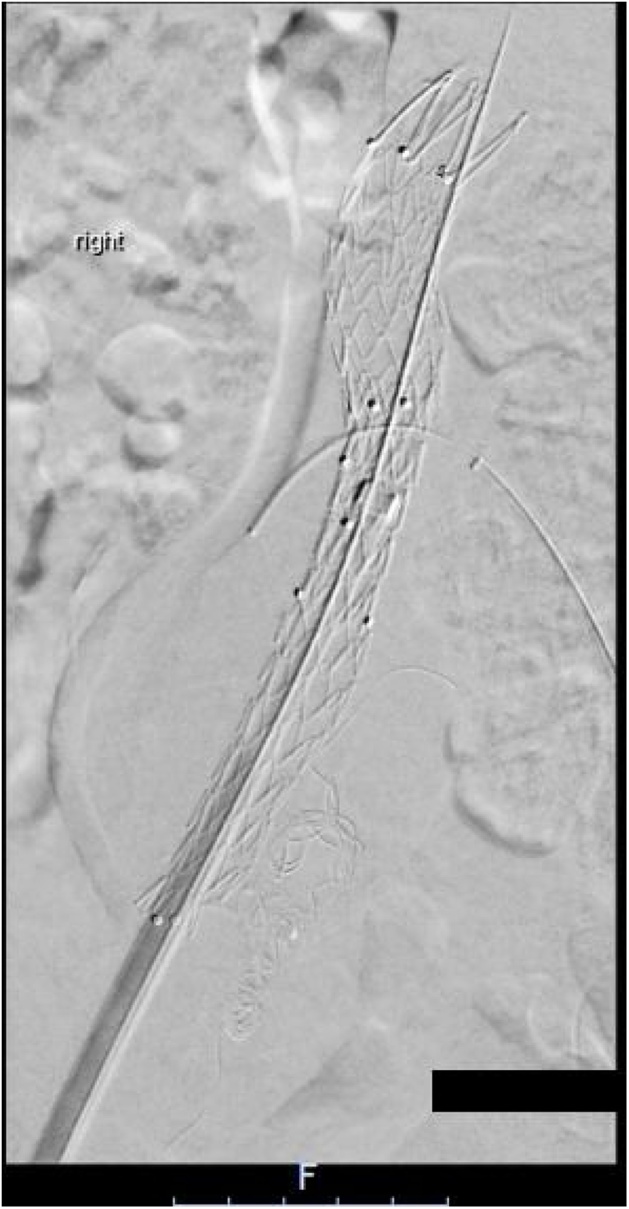
Final Deployment of Aorto-uni-iliac device.

**Fig. 4:**
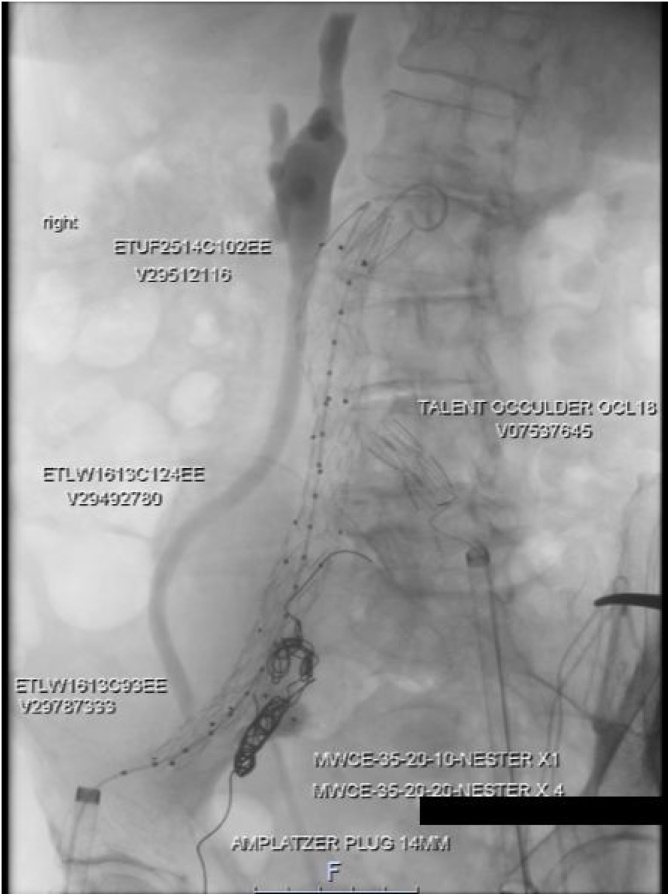
Completion Angiogram, showing Right Aorto-Uni-Iliac Device, Left Common Iliac Artery Occluder & embolised Right Internal Iliac Artery.

Subsequently via a Right common femoral artery open approach, the Endurant II Stent Graft System Aorto-Uni-Iliac device was deployed from the distal aorta to the right common iliac artery. The stent-graft limb was also extended to the right external iliac artery (Figs. 2 & 3 ). A left common femoral artery approach was used to occlude the left common iliac artery with a Talent occluder device. A good result was achieved with no complications on the final angiogram (Fig. 4).

In tandem, a right to left femoro-femoral crossover bypass graft was performed to maintain perfusion of both lower limbs.

This extra-abdominal approach with EVAR was considered safer for our patient, in view of her frailty and moderate anaesthetic risk.

She made a good post-operative recovery and was discharged home six days after intervention, to take 75 mg Aspirin Daily.

Her 30-day post EVAR CT Angiogram has not shown any Endoleak. She will follow our protocol for 12 monthly post-EVAR CT.

## 3 Discussion

As many as 70 % of patients are asymptomatic with an Isolated Iliac Artery Aneurysm (IIAA). This is likely due to the anatomical position of the Aneurysm being deep within the pelvis. If symptoms are present they can include Abdominal pain, an abdominal mass or other non- specific symptoms such as nausea, constipation or syncope. Urological Compressive symptoms have also been reported. These symptoms are often attributed to other disease processes hence the majority of IIAA are identified incidentally, often on radiological imaging. Because of this, diagnosis can be delayed and the aneurysm can be discovered at a significant size, the average being 5−6 cm [3].

Of course, a serious life-threatening presentation is rupture of the aneurysm. The size of the aneurysm at rupture is variable and can be difficult to measure but has been reported to be from 6 cm. There is a high associated risk of morbidity and mortality with ruptured aneurysm. Emergency interventional repair carries a mortality rate as high as 40–60 %. In comparison, the elective repair mortality rate is reported to be <11 % [1]. Based on studies detailing the progression of aneurysms, various Vascular Societies advise referral to a vascular specialist once a CIAA has reached a diameter of 3 cm [5]. It is believed that below this threshold the risk of rupture is low and outweighed by the risks of intervention. In practice, in the UK, most vascular surgeons would consider intervention until the diameter of the aneurysm was 4 cm, in an appropriate patient [6].

The choice of intervention has changed and developed over the years. Less invasive, endovascular techniques are superceding open repair. Advancement and availability of endovascular techniques make them an appealing strategy for elderly patients with multiple co-morbidities [7,8].

EVAR with Aorto-Uni-Iliac Device is a less time consuming and simpler procedure than bifurcated endovascular repair [9] in an urgent and elective setting. An important step in this procedure is the femoro-femoral bypass graft. Although this necessitates an additional extra-anatomic prosthetic graft the AUI configuration can be utilised in isolated common iliac artery aneurysm. Studies have demonstrated good medium and long term patency of the femoro-femoral graft [9].

**Fig. 5:**
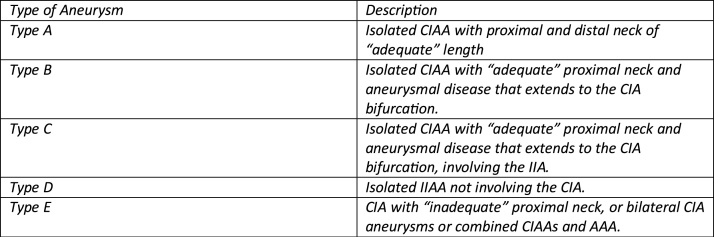
CIA- Common Iliac Artery, CIAA –Common Iliac Artery Aneurysm, IIA- Internal Iliac Artery, IIAA – Internal Iliac Artery Aneurysm, AAA- Abdominal Aorta Aneurysm.

The exact treatment option for CIAA will be based on the anatomical morphology and location of the aneurysm. Several classifications have been developed, which also take into account involvement of the Aorta and Internal Iliac Artery. A classification is detailed below (Fig. 5) [10,11].

In the case of our patient, for a Type C CIAA, a hybrid procedure was adopted.

Hybrid Theatres are allowing the integration of radiology and surgery engendering a collaborative approach to patient care [12]. This makes a hybrid operation an exciting opportunity to manage patients with large common iliac artery aneurysm and those with multiple co-morbidities for whom an open surgical approach is high risk.

Except for a few isolated case studies and reports [[13], [14], [15]] there is little literature detailing a hybrid technique that we used for our patient, in the management of unruptured isolated large common iliac artery aneurysm.

## 4 Conclusion

Our patient with an incidental finding of 8 cm Isolated Right Common Iliac Aneurysm underwent an urgent and safe hybrid procedure with an excellent outcome.

Even though non pulsatile, a significant mass in the lower abdomen, should also prompt the differential diagnosis of an aneurysm.

## Funding

No funding.

## Ethical approval

Patient’s consent sought for case report.

This is not a research study and so ethical approval was not applicable.

## Consent

Patient’s written and signed consent was obtained prior to submission.

## Author contribution

Case concept, Design – Mr Muhammad Asad Rahi & Dr Krishna Venigalla & Dr Maria Asad.

Image interpretation – Dr Krishna Venigalla.

Procedure performed by Mr Muhammad Asad Rahi & Dr Krishna Venigalla.

Paper Abstract and Discussion – written by Dr Maria Asad.

## Registration of research studies

According to research registry “We do not register case reports that are not first-in-man or animal studies”. Thus Not Applicable.

## Guarantor

Mr Muhammad Asad Rahi.

## Provenance and peer review

Not commissioned, externally peer-reviewed.

## Declaration of Competing Interest

No conflicts of Interest.
